# Charge effect on the photoinactivation of Gram-negative and Gram-positive bacteria by cationic *meso*-substituted porphyrins

**DOI:** 10.1186/1471-2180-9-70

**Published:** 2009-04-15

**Authors:** Eliana Alves, Liliana Costa, Carla MB Carvalho, João PC Tomé, Maria A Faustino, Maria GPMS Neves, Augusto C Tomé, José AS Cavaleiro, Ângela Cunha, Adelaide Almeida

**Affiliations:** 1 Department of Biology & CESAM, University of Aveiro, 3810-193 Aveiro, Portugal; 2 Department of Chemistry & QOPNA, University of Aveiro, 3810-193 Aveiro, Portugal

## Abstract

**Background:**

In recent times photodynamic antimicrobial therapy has been used to efficiently destroy Gram (+) and Gram (-) bacteria using cationic porphyrins as photosensitizers. There is an increasing interest in this approach, namely in the search of photosensitizers with adequate structural features for an efficient photoinactivation process. In this study we propose to compare the efficiency of seven cationic porphyrins differing in *meso*-substituent groups, charge number and charge distribution, on the photodynamic inactivation of a Gram (+) bacterium (*Enterococcus faecalis*) and of a Gram (-) bacterium (*Escherichia coli*). The present study complements our previous work on the search for photosensitizers that might be considered good candidates for the photoinactivation of a large spectrum of environmental microorganisms.

**Results:**

Bacterial suspension (10^7 ^CFU mL^-1^) treated with different photosensitizers concentrations (0.5, 1.0 and 5.0 μM) were exposed to white light (40 W m^-2^) for a total light dose of 64.8 J cm^-2^. The most effective photosensitizers against both bacterial strains were the Tri-Py^+^-Me-PF and Tri-Py^+^-Me-CO_2_Me at 5.0 μM with a light fluence of 64.8 J cm^-2^, leading to > 7.0 log (> 99,999%) of photoinactivation. The tetracationic porphyrin also proved to be a good photosensitizer against both bacterial strains. Both di-cationic and the monocationic porphyrins were the least effective ones.

**Conclusion:**

The number of positive charges, the charge distribution in the porphyrins' structure and the *meso*-substituent groups seem to have different effects on the photoinactivation of both bacteria. As the Tri-Py^+^-Me-PF porphyrin provides the highest log reduction using lower light doses, this photosensitizer can efficiently photoinactivate a large spectrum of environmental bacteria. The complete inactivation of both bacterial strains with low light fluence (40 W m^-2^) means that the photodynamic approach can be applied to wastewater treatment under natural light conditions which makes this technology cheap and feasible in terms of the light source.

## Background

Environmental contamination from domestic and industrial waste discharges has become a major public health concern. Wastewater treatment processing includes a final disinfection stage which eliminates pathogenic microorganisms (bacteria, virus and protozoa). Water disinfection can be achieved using chlorine, chlorine dioxide, hypochlorite, ozone or ultraviolet radiation. Although very efficient against a large range of microorganisms, the implementation of these solutions for wastewater treatment has been limited by environmental factors, namely the formation of toxic by-products from chorine [1], or by economic factors, as ultraviolet radiation and ozone treatment that are very expensive options to apply. Thus, as water reuse may be a way to cope with low water availability [2] in densely populated areas, more convenient and inexpensive technologies of water disinfection are needed [3].

Photodynamic antimicrobial therapy has recently been used to efficiently destroy microorganisms. This technique combines a photosensitizer (PS), typically a porphyrin or a phthalocyanine derivative with light and oxygen [4] leading to the formation of cytotoxic species (singlet oxygen and free radicals) that destroy those microorganisms [4]. This technique has been shown to be effective *in vitro *against bacteria (including drug-resistant strains), yeasts, viruses and protozoa [4,5]. Recent studies have shown that photoinactivation (PI) of bacteria in drinking [6] and residual waters [2,7] is possible under solar radiation. Bonnett et al. (2006) used a porphyrin and a phthalocyanine immobilized on a polymeric membrane of chitosan in a model reactor of water disinfection [6]. The recovery and reuse of immobilized PS opens the possibility to apply the photodynamic process in a real waste treatment system, avoiding the PS release and the contamination of water effluents [6,7].

In the last decade, several studies have used tetrapyrrolic derivatives as PS in order to assess the PI efficiency against Gram-negative [Gram (-)] and Gram-positive [Gram (+)] bacteria [2,8]. It has been well documented that neutral PS (porphyrins and phthalocyanines) efficiently destroy Gram (+) bacteria but are not able to photoinactivate Gram (-) bacteria [9-12]. However, many of these PS can become effective against Gram (-) bacteria if they are co-administrated with outer membrane disrupting agents such as CaCl_2_, EDTA or polymixin B nonapeptide [13,14] that are able to promote electrostatic repulsion with destabilization of the structure of the cell wall. This allows significant concentrations of the PS to penetrate the cytoplasmic membrane which can be photosensitized after light activation of the PS [15-19].

Porphyrins can be transformed into cationic entities through the insertion of positively charged substituents in the peripheral positions of the tetrapyrrole macrocycle that affect the kinetics and extent of binding with microbial cells [20]. The hydrophobicity degree of porphyrins can be modulated by either the number of cationic moieties (up to four in *meso*-substituted porphyrins) or by the introduction of hydrocarbon chains of different length on the amino nitrogens [20]. It has been reported that cationic porphyrin derivatives are able to induce the photoinactivation of Gram (+) and Gram (-) bacteria [2,11,21-23] and some studies have compared the efficiency of synthetic *meso*-substituted cationic porphyrins with different charge distribution (tetra-, tri-, di- or monocationic) [8,22-25]. However, results differ. Studies have demonstrated that tetracationic porphyrins are efficient PS against both Gram (+) and Gram (-) bacteria on visible light [22]; that some di- and tricationic porphyrins were more efficient than tetracationic ones, both against a Gram (+) strain and two Gram (-) strains [23]; and that a dicationic porphyrin as well as two tricationic porphyrins having a trifluoromethyl group were powerful photosensitizing agents against *Escherichia coli *[25].

Reviewing the literature, it can be said that there are some factors which increase the amphiphilic character of the porphyrins: the asymmetric charge distribution at the peripheral position of the porphyrin, cationic charges combined into different patterns with highly lipophilic groups (e.g., trifluoromethyl groups), the introduction of aromatic hydrocarbon side groups and the modulation of the number of positive charges on the PS [8,21,24,26-29]. This increase in the amphiphilic character of the PS seems to enhance its affinity for bacteria which improves its accumulation in the cells [25,27] and is accompanied by an increase in the photocytotoxic activity [24].

The aim of this study was to compare the efficiency of seven cationic porphyrins differing in *meso*-substituent groups, charge number and charge distribution, on the photodynamic inactivation of a Gram (+) bacterium (*Enterococcus faecalis*) and a Gram (-) bacterium (*Escherichia coli*). The choice of these porphyrins was based on the following facts: positive charges are required when the aim is to photoinactivate both Gram bacteria; these porphyrins are functionalized with groups that allow further immobilization on solid matrixes; previous studies performed in our laboratory showed that some of the selected porphyrins are efficient PS against other microorganisms such as sewage bacteriophage [30], bacterial endospores [31], sewage faecal coliforms [7] and recombinant bioluminescent *E. coli *[32]. The present study complements our previous work on the search for PS to be considered as good candidates for the photoinactivation of a large spectrum of environmental microorganisms.

The tetracationic porphyrin (Tetra-Py^+^-Me), extensively studied in bacterial and viral PI, was tested making it possible to evaluate the efficiency of the photodynamic process.

## Results

**Figure 1:**
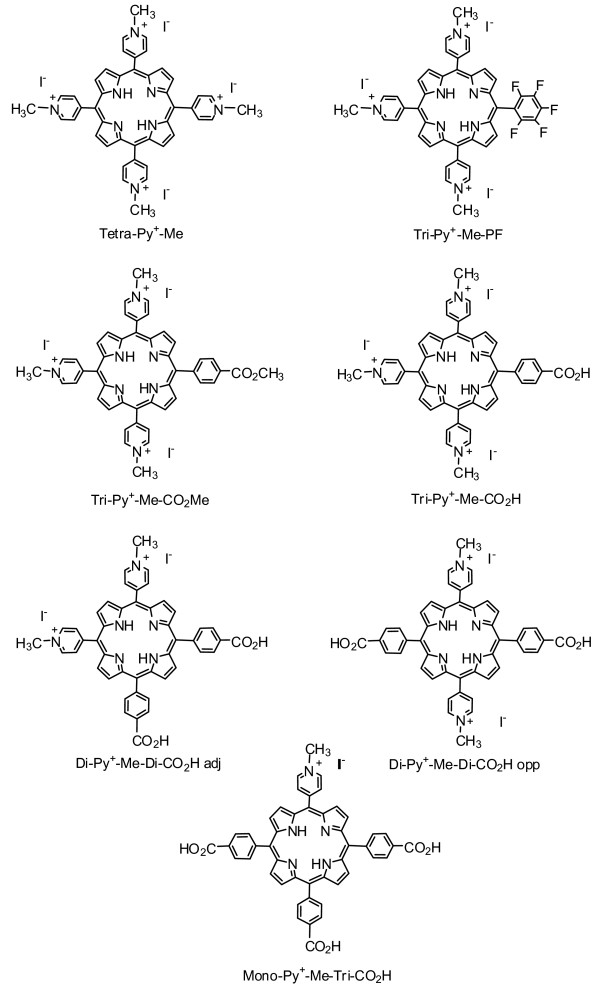
**Cationic porphyrin derivatives**. Structure of the seven cationic porphyrin derivatives used for photoinactivation of *E. faecalis *and *E. coli*.

We have tested the photocytotoxicity of seven *meso*-substituted cationic porphyrin derivatives (Fig. 1) differing in *meso*-substituent groups, charge number and charge distribution against *E. coli *and *E. faecalis*. All the new porphyrins were fully characterized by spectroscopic data and showed UV-Vis spectra of "Etio" type, typical of this type of derivatives. The efficiency of the PS was evaluated based on the determination of the number of viable colony forming units (CFU) per millilitre.

### Photodynamic inactivation of bacterial cells

**Figure 2:**
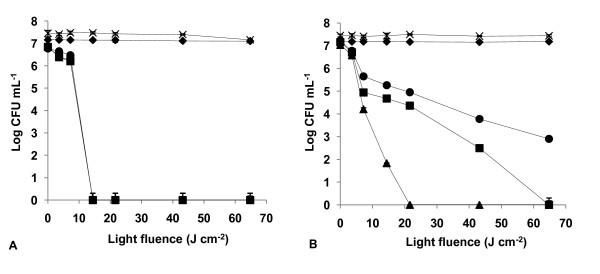
**Bacterial photoinactivation with Tri-Py^+^-Me-PF**. Survival curves of *E. faecalis *(A) and *E. coli *(B) (~10^7 ^CFU mL^-1^) incubated with porphyrin Tri-Py^+^-Me-PF and exposed to PAR light for different light doses. Light control (cross), dark control (filled diamond), 0.5 μM (filled circle), 1.0 μM (filled square), 5.0 μM (filled triangle). Values represent the mean of two independent experiments; error bars indicate the standard deviation.

**Figure 3:**
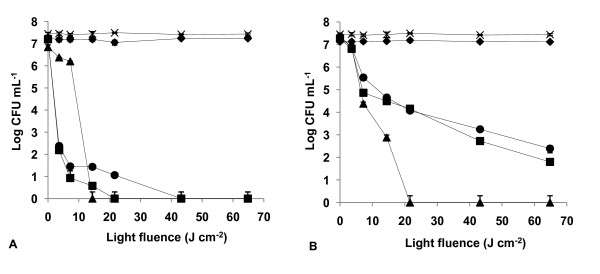
**Bacterial photoinactivation with Tri-Py^+^-Me-CO_2_Me**. Survival curves of *E. faecalis *(A) and *E. coli *(B) (~10^7 ^CFU mL^-1^) incubated with porphyrin Tri-Py^+^-Me-CO_2_Me and exposed to PAR light for different light doses. Light control (cross), dark control (filled diamond), 0.5 μM (filled circle), 1.0 μM (filled square), 5.0 μM (filled triangle). Values represent the mean of two independent experiments; error bars indicate the standard deviation.

**Figure 4:**
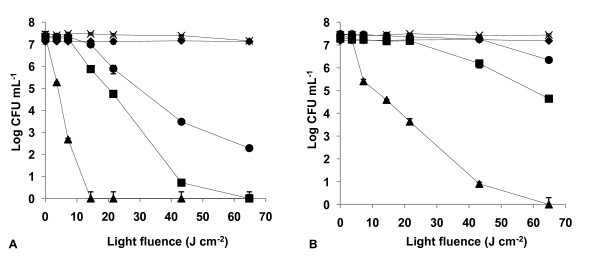
**Bacterial photoinactivation with Tetra-Py^+^-Me**. Survival curves of *E. faecalis *(A) and *E. coli *(B) (~10^7 ^CFU mL^-1^) incubated with porphyrin Tetra-Py^+^-Me and exposed to PAR light for different light doses. Light control (cross), dark control (filled diamond), 0.5 μM (filled circle), 1.0 μM (filled square), 5.0 μM (filled triangle). Values represent the mean of two independent experiments; error bars indicate the standard deviation.

**Figure 5:**
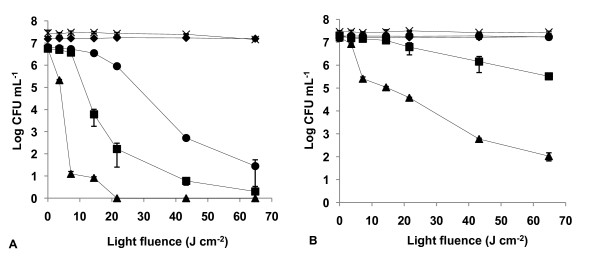
**Bacterial photoinactivation with Tri-Py^+^-Me-CO_2_H**. Survival curves of *E. faecalis *(A) and *E. coli *(B) (~10^7 ^CFU mL^-1^) incubated with porphyrin Tri-Py^+^-Me-CO_2_H and exposed to PAR light for different light doses. Light control (cross), dark control (filled diamond), 0.5 μM (filled circle), 1.0 μM (filled square), 5.0 μM (filled triangle). Values represent the mean of two independent experiments; error bars indicate the standard deviation.

**Figure 6:**
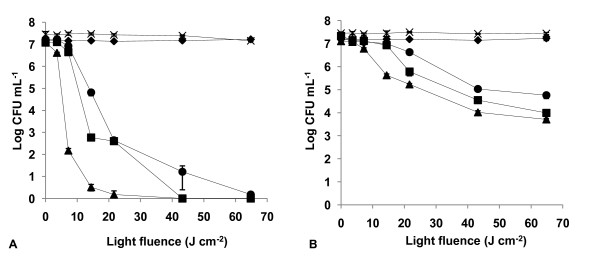
**Bacterial photoinactivation with Di-Py^+^-Me-Di-CO_2_H *adj***. Survival curves of *E. faecalis *(A) and *E. coli *(B) (~10^7 ^CFU mL^-1^) incubated with porphyrin Di-Py^+^-Me-Di-CO_2_H *adj *and exposed to PAR light for different light doses. Light control (cross), dark control (filled diamond), 0.5 μM (filled circle), 1.0 μM (filled square), 5.0 μM (filled triangle). Values represent the mean of two independent experiments; error bars indicate the standard deviation.

**Figure 7:**
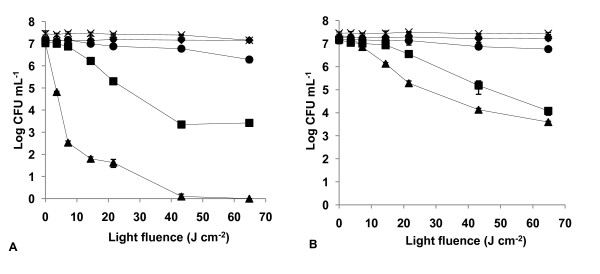
**Bacterial photoinactivation with Di-Py^+^-Me-Di-CO_2_H *opp***. Survival curves of *E. faecalis *(A) and *E. coli *(B) (~10^7 ^CFU mL^-1^) incubated with porphyrin Di-Py^+^-Me-Di-CO_2_H *opp *and exposed to PAR light for different light doses. Light control (cross), dark control (filled diamond), 0.5 μM (filled circle), 1.0 μM (filled square), 5.0 μM (filled triangle). Values represent the mean of two independent experiments; error bars indicate the standard deviation.

**Figure 8:**
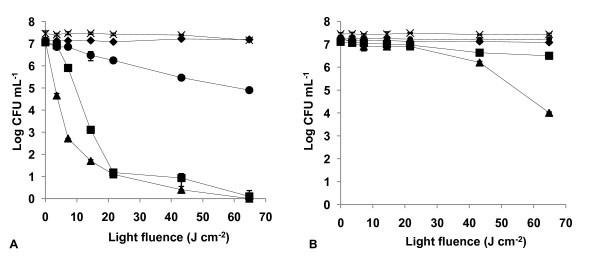
**Bacterial photoinactivation with Mono-Py^+^-Me-Tri-CO_2_H**. Survival curves of *E. faecalis *(A) and *E. coli *(B) (~10^7 ^CFU mL^-1^) incubated with porphyrin Mono-Py^+^-Me-Tri-CO_2_H and exposed to PAR light for different light doses. Light control (cross), dark control (filled diamond), 0.5 μM (filled circle), 1.0 μM (filled square), 5.0 μM (filled triangle). Values represent the mean of two independent experiments; error bars indicate the standard deviation.

The results of light and dark controls (Figs. 2, 3, 4, 5, 6, 7 and 8) showed that the viability of *E. coli *and *E. faecalis *is neither affected by irradiation itself (light control) nor by any of the PS tested in the dark (dark control) using the highest concentration studied (5.0 μM). In these controls ~7.2 log CFU mL^-1 ^is maintained during all experimental period. This indicates that the reduction obtained in cell viability after irradiation of the treated samples is due to the photosensitizing effect of the porphyrin.

The three tricationic porphyrin derivatives used were the most efficient PS against *E. faecalis *(~7 log survivors reduction with 5.0 μM) and demonstrated no significant difference in the photoinactivation of this strain (*p *> 0.05, ANOVA). However, Tri-Py^+^-Me-PF showed the most rapid decrease on *E. faecalis *survival causing a drop of ~6.80 log, after a light fluence of 14.4 J cm^-2 ^(*p *> 0.05, ANOVA), for each of the three concentrations tested (Fig. 2A).

The most efficient PS against *E. coli *were Tri-Py^+^-Me-PF and Tri-Py^+^-Me-CO_2_Me (*p *> 0.05, ANOVA) which caused more than a 7 log survivors reduction with 5.0 μM and after a light fluence of 21.6 J cm^-2 ^(Figs. 2B and 3B).

As expected, Tetra-Py^+^-Me was also a good PS against both bacteria, but it was not as efficient as the previous tricationic porphyrins (*p *< 0.05, ANOVA) for *E. faecalis*. In this case, the Tetra-Py^+^-Me caused a drop of 7.35 log, after a light fluence of 14.4 J cm^-2 ^at 5.0 μM (Fig. 4A). At lower concentrations 1.0 μM and 0.5 μM, and a light fluence of 64.8 J cm^-2 ^it caused a 7.33 log (99.77%) and a 5.07 log (93.23%) reduction, respectively. Against *E. coli*, this PS caused a 7.50 log reduction in survivors following a long irradiation period (64.8 J cm^-2 ^at a concentration of 5.0 μM) (Fig. 4B).

The tricationic porphyrin Tri-Py^+^-Me-CO_2_H was less effective for *E. coli *than the other two tricationic porphyrins (*p *< 0.05, ANOVA) (Fig. 5B). The best result (5.18 log reduction) was attained at a concentration of 5.0 μM and with a light fluence of 64.8 J cm^-2 ^(*p *= 1.000, ANOVA). This PS was less effective than Tetra-Py^+^-Me (*p *< 0.05, ANOVA), except for the concentration of 1.0 μM (*p *= 0.128, ANOVA).

The photoinactivation patterns for both dicationic porphyrins were not statistically different for *E. faecalis *at 1.0 and 5.0 μM (*p *> 0.05, ANOVA). However, at 0.5 μM there was a 7.03 log reduction with Di-Py^+^-Me-Di-CO_2_H *adj *compared with a 0.88 log reduction with Di-Py^+^-Me-Di-CO_2_H *opp *after 64.8 J cm^-2 ^of light exposure (Figs. 6A and 7A). ANOVA demonstrates that Di-Py^+^-Me-Di-CO_2_H *adj *was more effective than Di-Py^+^-Me-Di-CO_2_H *opp *at 0.5 μM of PS (*p *= 0.000, ANOVA). These dicationic porphyrins showed significant differences on the PI patterns against *E. coli *both at 0.5 μM and 5.0 μM (*p *< 0.05, ANOVA), with Di-Py^+^-Me-Di-CO_2_H *adj *as the most efficient. At 0.5 μM and 64.8 J cm^-2 ^of light dose produced a > 2.0 log decrease of cell inactivation. At the concentration of 5.0 μM the Di-Py^+^-Me-Di-CO_2_H *adj *and the Di-Py^+^-Me-Di-CO_2_H *opp *caused a similar survivors reduction (> 3.0 log) after a light fluence of 64.8 J cm^-2 ^(Fig. 6B and 7B).

Overall, the PI pattern against *E. faecalis *with Mono-Py^+^-Me-Tri-CO_2_H at 1.0 and 5.0 μM was not significantly different from Di-Py^+^-Me-Di-CO_2_H *adj *nor from Di-Py^+^-Me-Di-CO_2_H *opp *(*p *> 0.05, ANOVA). The comparison between Mono-Py^+^-Me-Tri-CO_2_H and Di-Py^+^-Me-Di-CO_2_H *opp *revealed that at 0.5 μM and long irradiation periods Mono-Py^+^-Me-Tri-CO_2_H showed more PI activity than Di-Py^+^-Me-Di-CO_2_H *opp*: 2.16 log survivors reduction versus 0.88 log survivors reduction, respectively (*p *= 0.000, ANOVA) (Figs. 7A and 6A). This means that monocationic porphyrin is more effective than the dicationic *opp *porphyrin, when the lower concentration of PS is used on this strain. Against *E. coli*, this monocationic porphyrin was only significantly different from Di-Py^+^-Me-Di-CO_2_H *opp *(*p *= 0.000, ANOVA), at concentrations of 0.5 μM (Fig. 8B). The major inactivation observed (3.28 log) with Mono-Py^+^-Me-Tri-CO_2_H resulted at a concentration of 5.0 μM and after a light fluence of 64.8 J cm^-2^.

### Singlet oxygen generation studies and partition coefficients

**Figure 9:**
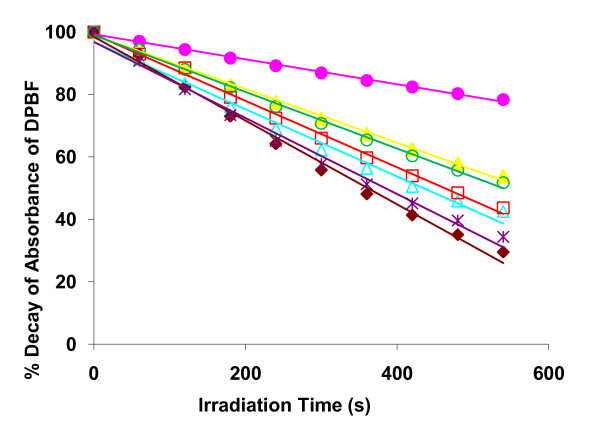
**Photodecomposition of DPBF**. Photodecomposition of DPBF in DMF/H_2_O (9:1) by singlet oxygen generated by the photosensitizers after irradiation with white light filtered through a cut-off filter for wavelengths < 540 nm (9 mW cm^-2^). Tetra-Py^+^-Me (pink filled circle), Tri-Py^+^-Me-PF (yellow filled triangle), Tri-Py^+^-Me-CO_2_Me (light blue open triangle), Tri-Py^+^-Me-CO_2_H (red open square), Di-Py^+^-Me-Di-CO_2_H *opp *(brown filled diamond), Di-Py^+^-Me-Di-CO_2_H *adj *(violet star), Mono-Py^+^-Me-Tri-CO_2_H (green open circle).

The ability of these cationic porphyrin derivatives to generate singlet oxygen, the basis of the photoinactivation process, was qualitatively evaluated by monitoring the photodecomposition of 1,3-diphenylisobenzofuran (DPBF). The results, summarized in Fig. 9 and Table 1, show that the DPBF photodegradation was highly enhanced in the presence of the PS. The tri-, di- and monocationic porphyrin derivatives with slopes varying between 0.086–0.134 (the slope is proportional to the rate of production of singlet oxygen) showed to be, under the same experimental conditions, more efficient than Tetra-Py^+^-Me (slope 0.040) considered a good singlet oxygen producer [2,22,33].

Since partition coefficients are difficult to measure in living systems, they are usually obtained in vitro using a hydrophobic and hydrophilic phase. The partition coefficient (P) is the ratio of the solubility of a solute in the organic and aqueous phases. In this case, in order to obtain reproducible results, the partition coefficients (log P_B/W_) were determined in a butan-1-ol/water system [22,34,35]. The results (Table 1) indicate that the most hydrophilic PS is Tetra-Py^+^-Me and the most hydrophobic one is the Tri-Py-Me^+^-CO_2_Me. The log P_B/W _values of the porphyrin derivatives containing the free carboxylic groups showed that the Tri-Py-Me^+^-CO_2_H and Di-Py^+^-Me-Di-CO_2_H *adj *are more hydrophilic (~-0.9) than Di-Py^+^-Me-Di-CO_2_H *opp *and Mono-Py^+^-Me-Tri-CO_2_H (~-0.3). These results are consistent with the expected polarity of these molecules. The more amphiphilic PS is Tri-Py^+^-Me-PF with a log P_B/W _value of -0.17.

**Table 1:** Rate of ^1^O_2 _production and partition coefficients

Porphyrin Derivatives	Slope	**Log P**_B/W_
Tetra-Py^+^-Me	0.040	-1.97
Tri-Py^+^-Me-PF	0.086	-0.17
Tri-Py-Me^+^-CO_2_Me	0.113	1.91
Tri-Py^+^-Me-CO_2_H	0.106	-0.95
Di-Py^+^-Me-Di-CO_2_H *adj*	0.122	-0.98
Di-Py^+^-Me-Di-CO_2_H *opp*	0.134	-0.31
Mono-Py^+^-Me-Tri-CO_2_H	0.091	-0.29

Values of slope of the plots of absorbance of DPBF in DMF/water (9:1) versus ilumination time and butan-1-ol/water partition coefficients (log P_B/W_) for each photosensitizer.

## Discussion

According to the results obtained, all the seven *meso*-substituted cationic porphyrins have shown to be very good singlet oxygen generators. However, this study shows that the bacterial PI process of both Gram (+) and Gram (-) bacteria is dependent on the number of positive charges, charge distribution and nature of *meso*-substituent groups present in the macrocycle periphery.

The cationic porphyrin derivatives selected induce direct PI of Gram (+) and also of Gram (-) bacteria. This type of porphyrins is able to inactivate directly the Gram (-) cells without the presence of additives. The positive charge on the PS molecule promotes a tight electrostatic interaction with negatively charged sites at the outer surface of the bacterial cells, increasing the efficiency of the PI process [19,22,23,36]. All porphyrins in this study were effective PS against Gram (+) strain *E. faecalis *achieving ~7 log (more than 99.999%) reduction on cell survival after light exposure at the highest concentration (5.0 μM). The PI process against the Gram (-) strain, *E. coli*, was efficient (~7.50 log survivors reduction) with Tri-Py^+^-Me-PF, Tri-Py^+^-Me-CO_2_Me and Tetra-Py^+^-Me at 5.0 μM and after a light fluence of 21.6–64.8 J cm^-2^. The reduction in cell survival for that maximum light dose and concentration (64.8 J cm^-2 ^and 5.0 μM) is much lower with Tri-Py^+^-Me-CO_2_H (5.18 log, 99.998%), Di-Py^+^-Me-Di-CO_2_H *opp *(3.77 log, 99.98%), Di-Py^+^-Me-Di-CO_2_H *adj *(3.40 log, 99.96%) and Mono-Py^+^-Me-Tri-CO_2_H (3.28, 99.93%).

The PI patterns of both bacterial strains with all seven porphyrins were different. In general, against *E*. *faecalis*, the efficiency of PS followed the order: Tri-Py^+^-Me-PF = Tri-Py^+^-Me-CO_2_Me = Tri-Py^+^-Me-CO_2_H > Di-Py^+^-Me-Di-CO_2_H *adj *> Tetra-Py^+^-Me > Mono-Py^+^-Me-Tri-CO_2_H > Di-Py^+^-Me-Di-CO_2_H *opp*. Against *E. coli*, the order is Tri-Py^+^-Me-PF = Tri-Py^+^-Me-CO_2_Me > Tetra-Py^+^-Me > Tri-Py^+^-Me-CO_2_H > Di-Py^+^-Me-Di-CO_2_H *adj *> Di-Py^+^-Me-Di-CO_2_H *opp *> Mono-Py^+^-Me-Tri-CO_2_H. The porphyrins with three and four positive charges were the most effective PS against both bacterial strains. Some of these compounds, besides being highly effective against both bacteria strains, were also able to efficiently photoinactivate sewage faecal coliforms [7], sewage bacteriophage [30] and bacterial endospores [31]. In this study, Tri-Py^+^-Me-PF and Tri-Py^+^-Me-CO_2_Me were even more efficient than Tetra-Py^+^-Me. It was expected that by increasing the number of positive charges the cell killing should also increase. In fact, some studies have showed a high rate of bacterial inactivation with tri- and tetracationic porphyrins compared with di- and monocationic ones [24,25]. However, other studies have reported contradictory results: Merchat et al. (1996) concluded that the number of charges does not affect the activity of the PS against both bacterial Gram types [23]. Caminos et al. (2006) showed that the photodynamic activity of a tricationic porphyrin combined with trifluoromethyl group was higher for an *E. coli *strain than the one observed with the corresponding tetracationic porphyrin [24]. Banfi et al. (2006) also concluded that a dicationic porphyrin was more efficient than the corresponding tetracationic derivatives against Gram (+) *Staphylococcus aureus *and Gram (-) *E. coli *and *Pseudomonas aeruginosa *[21]. However, our results suggest that the number of positive charges affects the PI process. Two of the tricationic PS are the most efficient ones, although they have quite different partition coefficients.

Comparing the photoinactivation rate of Tri-Py^+^-Me-PF and Tri-Py^+^-Me-CO_2_Me with the photoinactivation rate of tetracationic Tetra-Py^+^-Me, the results suggest that a high number of positive charges and a hydrophilic character can decrease the PI efficiency, as shown by other studies (Jori, personal communication). On the other hand, the *meso*-substituent groups can play an important role on bacterial PI process. In fact, it has been shown that positive charges combined with highly lipophilic groups might increase the amphiphilic character of the PS, enhancing its affinity to bacteria [25,27], and consequently increasing the photocytotoxic activity [24]. However, in the present study no direct correlation could be established between the PI pattern and the partition coefficients of the PS. Probably, other interactions, not accounted by log P_B/W_, such as the combined effect of the cationic charge and the amphiphilic character of the macrocycle is responsible for the photodynamic efficiency [19,20,34].

In our case, the results obtained with Tri-Py^+^-Me-PF and Tri-Py^+^-Me-CO_2_Me against *E. coli *were significantly different (*p *= 0.000, ANOVA) from those obtained with the other tricationic porphyrin Tri-Py^+^-Me-CO_2_H. Tri-Py^+^-Me-PF, and Tri-Py^+^-Me-CO_2_Me caused a reduction below the detectable limits (~7 log) after a light dose of 21.6 J cm^-2 ^on *E. coli *while Tri-Py^+^-Me-CO_2_H produced only a ~5 log survivors reduction after 64.8 J cm^-2^. A possible explanation for this behaviour can be the presence of the acid group in the Tri-Py^+^-Me-CO_2_H porphyrin. This acid group can be ionized when dissolved in PBS buffer and the global charge of the porphyrin decreases and, consequently, the efficiency of inactivation decreases. On the other hand, the Tri-Py^+^-Me-CO_2_Me, that has the acid group protected, shows a significantly higher (*p *< 0.000, ANOVA) inactivation rate for *E. coli *than Tri-Py^+^-Me-CO_2_H. The results achieved with Tri-Py^+^-Me-PF and Tri-Py^+^-Me-CO_2_Me confirm that the nature of the *meso*-substituent groups appears to play an important role in bacterial inactivation, as already observed in similar studies [24,25,27].

The distribution of the charges on the sensitizer is another factor that influences the efficiency of the PI process. In this study, the pattern of inactivation by symmetric and asymmetric dicationic porphyrins was significantly different, although they both have a similar capacity of producing singlet oxygen. Di-Py^+^-Me-Di-CO_2_H *adj *showed a higher efficiency on the photoinactivation of *E. coli *than Di-Py^+^-Me-Di-CO_2_H *opp *at the lower (0.5 μM) and highest (5.0 μM) concentrations. On *E. faecalis*, Di-Py^+^-Me-Di-CO_2_H *adj *it is also significantly different from Di-Py^+^-Me-Di-CO_2_H *opp *only when the lower concentration (0.5 μM) is used (*p *= 0.000, ANOVA). These results are in accordance with Kessel el al. (2003) studies that reported the cell localization and photodynamic efficacy of two dicationic porphyrins on Murine L 1210 cells. The PS with the two charges in adjacent positions was five-fold more efficient than the one with the charges in opposite positions [37]. The two adjacent positive charges in the porphyrin macrocycle should result in a molecular distortion due to electrostatic repulsion. In contrast, the porphyrin with the two opposite positive charges is a much more symmetric molecule. The affinity of these asymmetric cationic molecules with cell structures has yet to be established, but it is thought to be a function of hydrophobicity factors, charge distribution or both [37].

The Mono-Py^+^-Me-Tri-CO_2_H was the most inefficient PS against *E. coli*, causing a 3.28 log reduction on this strain and only after a total light dose of 64.8 J cm^-2 ^(5.0 μM). This result is in agreement with previous studies where monocationic sensitizers were tested against Gram (-) bacteria [23,24].

## Conclusion

The results obtained in this study show that the cationic porphyrins having three and four charges are highly efficient PS against both bacterial strains. The distinct *meso*-substituent groups in the porphyrin structure seem to have different effects on PI. The Tri-Py^+^-Me-PF porphyrin provides the highest log reduction on cell survival using lower light doses. From this study and bearing in mind the development of efficient PS able to photoinactivate a large spectrum of environmental microorganisms, the Tri-Py^+^-Me-PF is the most promising PS. In addition, the PI of Gram (+) and also of Gram (-) bacteria using a higher bacterial density (10^7 ^CFU mL^-1^) than the levels present in wastewater (10^4^–10^5 ^CFU mL^-1^) ensures its efficiency.

Since this technology is to be used in the real context of a flow system and under solar light which is much more intense than the white light used in our studies (on average 456 W m^-2 ^considering winter and summer periods in the city of Aveiro), the time needed for the photodynamic inactivation to occur would be substantially shorter. Therefore, this photodynamic approach applied to wastewater treatment under natural light conditions makes this technology cheap and feasible in terms of light source. As the Tri-Py^+^-Me-PF has a pentafluorophenyl group that allows its immobilization on a solid support, the photoinactivation process can occur without the release of the PS to the water output, making this approach also environmentally-friendly.

## Methods

### Photosensitizers

5,10,15,20-tetrakis(1-methylpiridinium-4-yl)porphyrin tetra-iodide (Tetra-Py^+^-Me), 5-(pentafluorophenyl)-10,15,20-tris(1-methylpiridinium-4-yl)porphyrin tri-iodide (Tri-Py^+^-Me-PF), 5-(4-methoxicarbonylphenyl)-10,15,20-tris(1-methylpiridinium-4-yl)porphyrin tri-iodide (Tri-Py^+^-Me-CO_2_Me), 5-(4-carboxyphenyl)-10,15,20-tris(1-methylpiridinium-4-yl)porphyrin tri-iodide (Tri-Py^+^-Me-CO_2_H), 5,10-bis(4-carboxyphenyl)-15,20-bis(1-methylpiridinium-4-yl)porphyrin di-iodide (Di-Py^+^-Me-Di-CO_2_H *adj*), 5,15-bis(4-carboxyphenyl)-10,20-bis(1-methylpiridinium-4-yl)porphyrin di-iodide (Di-Py^+^-Me-Di-CO_2_H *opp*) and 5-(1-methylpiridinium-4-yl)-10,15,20-tris(4-carboxyphenyl)porphyrin iodide (Mono-Py^+^-Me-Tri-CO_2_H) (Fig. 1) were prepared in two steps. First, the neutral porphyrins were obtained from the Rothemund and crossed Rothemund reactions using pyrrole and the appropriate benzaldehydes (pyridine-4-carbaldehyde and pentafluorophenylbenzaldehyde or 4-formylbenzoic acid) at reflux in acetic acid and nitrobenzene ([38-40]. After being separated by column chromatography (silica), the pyridyl groups of each porphyrin were quaternized by reaction with methyl iodide. Porphyrin Tri-Py^+^-Me-CO_2_Me was obtained by esterification of the corresponding acid derivative with methanol/sulphuric acid followed by quaternization with methyl iodide. Porphyrins were purified by crystallization from chloroform-methanol-petroleum ether and their purities were confirmed by thin layer chromatography and by ^1^H NMR spectroscopy. The spectroscopic data was in accordance with the literature [38-40]. Stock solutions (500 μM) of each porphyrin in dimethyl sulfoxide were prepared by dissolving the adequate amount of the desired porphyrin in a known volume. The absorption spectral features of the PS were the following: [porphyrin] λ_max _nm (log ε); [Tetra-Py^+^-Me] in DMSO 425 (5.43), 516 (4.29), 549 (3.77), 588 (3.84), 642 (3.30); [Tri-Py^+^-Me-PF] in DMSO 422 (5.48), 485 (3.85), 513 (4.30), 545 (3.70), 640 (3.14); [Tri-Py^+^-Me-CO_2_Me] in H_2_O 420 (5.54), 518 (4.12), 556 (3.74), 583 (3.78), 640 (3.27); [Tri-Py^+^-Me-CO_2_H] in H_2_O 425 (5.40), 520 (4.24), 555 (3.90), 588 (3.82), 646 (3.34); [Di-Py^+^-Me-Di-CO_2_H *adj*] in H_2_O 425 (5.21), 521 (4.06), 557 (3.78), 590 (3.64), 648 (3.04); [Di-Py^+^-Me-Di-CO_2_H opp] in H_2_O 424 (5.40), 518 (4.16), 558 (3.94), 589 (3.69), 648 (3.58); [Mono-Py^+^-Me-Tri-CO_2_H] in butan-1-ol 425 (5.35), 520 (4.25), 553 (4.01), 591 (3.87), 649 (3.74). Selected data: [Di-Py^+^-Me-Di-CO_2_H *opp*] ^1^H-NMR: (300 MHz, DMSO-d6) δ 9.46 (4H, d, *J *6.6 Hz, 10,20-Ar-*m*-H), 8.99 – 9.05 (12H, m, 10,20-Ar-*o*- and β-H), 8.41 (4H, d, *J *8.0 Hz, 5,15-Ar-*m*-H), 8.30 (4H, d, *J *8.0 Hz, 5,15-Ar-*o*-H), 4.70 (6H, s, 2 × CH_3_), -2.99 (2H, s, NH). MS (MALDI-TOF) *m/z*: 734.2 (M-2I)^+^; [Di-Py^+^-Me-Di-CO_2_H *adj*] ^1^H-NMR: (300 MHz, DMSO-d6) δ 9.46 (4H, d, *J *6.7 Hz, 15,20-Ar-*m*-H), 8.92 – 9.12 (12H, m, 15,20-Ar-*o*- and β-H), 8.40 (4H, d, *J *8.2 Hz, 5,10-Ar-*m*-H), 8.30 (4H, d, *J *8.2 Hz, 5,10-Ar-*o*-H), 4.70 (6H, s, 2xCH_3_), -2.96 (2H, s, NH). MS (MALDI-TOF) *m/z*: 734.2 (M-2I)^+^; [Mono-Py^+^-Me-Tri-CO_2_H] ^1^H-NMR: (300 MHz, DMSO-d6) δ 9.44 (2H, d, *J *6.4 Hz, 20-Ar-*m*-H), 8.90 – 9.03 (10H, m, 20-Ar-*o*- and β-H), 8.30 – 8.40 (12H, m, 5,10,15-Ar-H), 4.69 (3H, s, CH_3_), -2.94 (2H, s, NH). MS (MALDI-TOF) *m/z*: 762.2 (M-I)^+^.

### Partition coefficients

The partition coefficients were determined at 22°C in butan-1-ol/water (log P_B/W_) according to the shake-flask method. Porphyrin derivatives were individually dissolved in water-saturated butan-1-ol to give the stock solution (absorbance ~0.8 at the Soret band). Then, in duplicate test vessels, different volumes of butan-1-ol-saturated water and stock porphyrin solution were added in order to get at least three different butan-1-ol/water volume ratio. Each vessel was vigorously vortexed and then centrifuged to allow phase separation and kept for equilibration at the test temperature for 2 hours before analysis. The absorbance at the Soret band was measured in both phases and the log P_B/W _determined using the relationship log P_B/W _= log (Abs_B _*V_W_/Abs_W _*V_B_), where Abs_W _and Abs_B _are the absorbances at the Soret band and V_W _and V_B _are the volumes of aqueous and butan-1-ol phases, respectively [35].

### Singlet oxygen generation studies

Stock solution of each porphyrin derivative at 0.1 mM in DMF: water (9:1) and a stock solution of 1,3-diphenylisobenzofuran (DPBF) at 10 mM in DMSO were prepared. The reaction mixture of 50 μM of DPBF and 0.5 μM of a porphyrin derivative in DMF water (9:1) in glass cells (2 mL) was irradiated with white light filtered through a cut-off filter of wavelength < 540 nm, at a fluence rate of 9.0 mW cm^-2^. During the irradiation period, the solutions were stirred at room temperature. The generation of singlet oxygen was followed by its reaction with DPBF. The breakdown of DPBF was monitored by measuring the decreasing of the absorbance at 415 nm at irradiation intervals of 1 min.

### Bacterial strains and growth conditions

*Escherichia coli *ATCC 13706 (USA) and *Enterococcus faecalis *ATCC 29212 (USA) were stored at 4°C in triptic soy agar (TSA, Merck). Before each assay the strains were grown aerobically for 24 hours at 37°C in 30 mL of triptic soy broth (TSB, Merck). An aliquot of this culture (240 μL) was aseptically transferred to 30 mL of fresh TSB medium and grown overnight at 37°C to reach an optical density (O.D._600_) of ~1.3, corresponding to ~10^8 ^cells mL^-1^.

### Experimental setup

The efficiency of the cationic porphyrins at different concentrations (0.5, 1.0 and 5.0 μM) was evaluated through quantification of the colonies of bacteria in laboratory conditions. Knowing that the inactivation of bacteria by cationic porphyrins is very sensitive to ionic strength [41], all the experiments were performed using the same conditions. Bacterial suspensions were prepared from bacterial cultures (~10^8 ^cells mL^-1^) which were diluted ten-fold in phosphate buffered saline, pH 7.4, to a concentration of ~10^7 ^CFU mL^-1^(100–1000 times higher than bacterial concentration in wastewater to ensure that when applied to the field most of similar bacteria were inactivated). In all the experiments, 49.5 mL of bacterial suspension were aseptically distributed in 600 mL acid-washed, sterilised glass beakers and the PS was added from the stock solution (500 μM in DMSO) to achieve final concentrations of 0.5, 1.0 and 5.0 μM. After the addition of the appropriate volume of porphyrin, beakers (total volume of 50 mL) were incubated during 10 minutes at 20–25°C, under stirring (100 rpm), covered with aluminium foil to avoid accidental light exposure.

Light and dark control experiments were carried out simultaneously. In the light controls, the bacterial suspension without PS was exposed to light irradiation. In the dark controls, the PS at the higher concentration (5.0 μM), was added to the beaker, containing the bacterial suspension, covered with aluminium foil to protect from light exposure. The controls also followed the pre-irradiation incubation protocol.

This photosensitization procedure was used for each of the seven PS tested and for both bacterial strains under investigation.

### Irradiation conditions

Following the pre-irradiation incubation period, all samples were exposed in parallel to white light (PAR radiation, 13 OSRAM 21 lamps of 18 W each, 380–700 nm) with a fluence rate of 40 W m^-2 ^(measured with a light meter LI-COR Model LI-250, Li-Cor Inc., USA), at 20–25°C for 270 minutes, under 100 rpm mechanical stirring.

### Bacterial quantification

A standard volume (1 mL) of undiluted and serially diluted of irradiated samples and controls were plated in duplicate in TSA medium at time 0 and after 15, 30, 60, 90, 180 and 270 minutes of light exposure. After 24 hours of incubation at 37°C in the dark, the number of colonies was counted. The dark control Petri plates were kept in the dark immediately after plating and during the incubation period. The assays for each concentration of each porphyrin and for each bacterial strain were done in duplicate and averaged. Data were presented by survival curves plotted as logarithmic bacterial reduction in log CFU mL^-1 ^versus light fluence in J cm^-2^. As previously stated, bactericidal activity was defined as a ≥ 3 log decrease (≥ 99,9%) in CFU mL^-1^, while bacteriostatic activity was defined as a <3 log (< 99,9%) decrease in CFU mL^-1 ^[42].

### Statistical analysis

Statistical analyses were performed by using SPSS (SPSS 15.0 for Windows, SPSS Inc., USA). Normal distributions were assessed by Kolmogorov-Smirnov test. The significance of both porphyrin derivatives and irradiation time on bacterial inactivation was assessed by two-way univariate analysis of variance (ANOVA) model with the Bonferroni post-hoc test. A value of p < 0.05 was considered significant.

## Abbreviations

(PS): Photosensitizer; (PI): Photoinactivation; [Gram (-)]: Gram-negative; [Gram (+)] bacteria: Gram-positive; (CFU): colony forming units; (DMSO): dimethyl sulfoxide; (Tetra-Py^+^-Me): 5,10,15,20-tetrakis(1-methylpiridinium-4-yl)porphyrin tetra-iodide; (Tri-Py^+^-Me-PF): 5-(pentafluorophenyl)-10,15,20-tris(1-methylpiridinium-4-yl)porphyrin tri-iodide; (Tri-Py^+^-Me-CO_2_Me): 5-(4-methoxicarbonylphenyl)-10,15,20-tris(1-methylpiridinium-4-yl)porphyrin tri-iodide; (Tri-Py^+^-Me-CO_2_H): 5-(4-carboxyphenyl)-10,15,20-tris(1-methylpiridinium-4-yl)porphyrin tri-iodide; (Di-Py^+^-Me-Di-CO_2_H *adj*): 5,10-bis(4-carboxyphenyl)-15,20-bis(1-methylpiridinium-4-yl)porphyrin di-iodide; (Di-Py^+^-Me-Di-CO_2_H *opp*): 5,15-bis(4-carboxyphenyl)-10,20-bis(1-methylpiridinium-4-yl)porphyrin di-iodide; (Mono-Py^+^-Me-Tri-CO_2_H): 5-(1-methylpiridinium-4-yl)-10,15,20-tris(4-carboxyphenyl)porphyrin iodide.

## Authors' contributions

EA carried out all the photoinactivation experiments with porphyrins, statistics and analyses of data and drafted the manuscript. CMBC, JPCT, MAFF, MGPMSN, ACT and JASC participated on the synthesis of porphyrins, purification process as well as structural characterization; performed the coefficient partition, singlet oxygen generation studies, and helped to draft the manuscript. AA has been involved in the coordination, conception, design of the study and helped to draft the manuscript. LC and AC participated in the design of the study, acquisition and interpretation of data, and also helped to draft the manuscript. All authors have read and approved the final manuscript.
